# Novel Specific Pyruvate Kinase M2 Inhibitor, Compound 3h, Induces Apoptosis and Autophagy through Suppressing Akt/mTOR Signaling Pathway in LNCaP Cells

**DOI:** 10.3390/cancers15010265

**Published:** 2022-12-30

**Authors:** Chunxue Jiang, Xiaodi Zhao, Taejoo Jeong, Ju Young Kang, Jae Hyeon Park, In Su Kim, Hyung Sik Kim

**Affiliations:** 1 School of Pharmacy, Sungkyunkwan University, Suwon 16419, Republic of Korea; 2 Department of Biopharmaceutical Convergence, Sungkyunkwan University, Suwon 16419, Republic of Korea

**Keywords:** PKM2 inhibitors, cell metabolism, prostate cancer, apoptosis, glycolysis

## Abstract

**Simple Summary:**

Prostate cancer is a serious threat to male health worldwide; however, chemotherapy remains an urgent problem because of resistance and insensitivity to androgen-deprivation therapy. Therefore, discovering novel molecules and chemotherapy can provide a new strategy. Pyruvate kinase M2 (PKM2) is not only a vital enzyme in regulating cancer glycolysis, but is also a transcription that regulates gene expression through nuclear translocation, so the targeting PKM2 can be a promising therapy for prostate cancer. In this experiment, we found a novel specific PKM2 inhibitor—compound **3h**—and conducted molecular and cellular experiments to evaluate its anticancer effect. Our results indicated that proliferation of LNCaP cells can be inhibited by compound **3h** through inducing apoptosis and autophagy, and inhibiting glycolysis and mitochondria respiration. Therefore, our results provide a new way of developing novel chemotherapy for prostate cancer.

**Abstract:**

Pyruvate kinase M2 (PKM2) is a key enzyme involved in the regulation of glycolysis. Although PKM2 is overexpressed in various tumor tissues, its functional role in cancer chemotherapy remains unexplored. In this study, we investigated the anticancer activity of a new PKM2 inhibitor, compound **3h**, through the cell metabolism and associated signaling pathways in prostate cancer cells. To evaluate the molecular basis of specific PKM2 inhibitors, the interactions of compounds **3h** and **3K** with the PKM2 protein were assessed via molecular docking. We found that, compared to compound **3K**, compound **3h** exhibited a higher binding affinity for PKM2. Moreover, compound **3h** significantly inhibited the pyruvate kinase activity and PKM2 expression. Cytotoxicity and colony formation assays revealed the potent anticancer activity of compound **3h** against LNCaP cells. Compound **3h** significantly increased the apoptotic and autophagic cell death in LNCaP cells. In addition, compound **3h** induced AMPK activation along with the inhibition of the mTOR/p70S6K pathway. Furthermore, compound **3h** significantly inhibited glycolysis and mitochondrial respiration, as determined by analyzing the extracellular acidification rate (ECAR) and oxygen consumption rate (OCR) production. Our results revealed that compound **3h** caused apoptotic and autophagic cell death in LNCaP cells by inhibiting cancer cell metabolism. Therefore, blocking glycolytic pathways using specific PKM2 inhibitors can target cancer cell metabolism in PKM2-overexpressed prostate cancer cells.

## 1. Introduction

Prostate cancer has the character of malignant growth metastasis, and prostate cancer with bone marrow metastasis, in particular, is a global medical problem with no efficient therapy. Prostate cancer is considered the second greatest threat to the health of American males, with 268,490 new cases and 34,500 deaths predicted to occur in 2022, according to American Cancer Society [1]. Sixty percent of patients with prostate cancer are diagnosed at the age of 65 or older; it is rarely diagnosed in individuals younger than 40 years [2]

**Figure 1:**
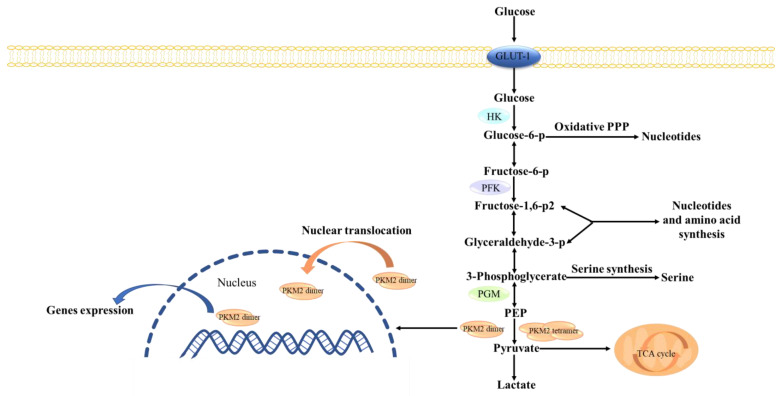
The tetramer and dimer of PKM2 regulate glycolysis and gene transcription in cancer cells. The active tetramer PKM2 is a glycolytic enzyme that transfers PEP into pyruvate, which enters mitochondria and joins in the TCA cycle. The low activity PKM2 dimer leads to the accumulation of glycolytic intermediates to support the biosynthetic precursors of proliferating cells. In addition, dimeric PKM2, as a transcription factor, translocates to the nucleus and activates and regulates transcription of certain genes. GLUT1: Glucose transporter 1, PPP: Pentose Phosphate Pathway, HK: Hexokinase, PFK: Phosphofructokinase 1, PGM: Phosphoglycerate mutase, PEP: Phosphoenolpyruvate. TCA cycle: Tricarboxylic acid cycle, PKM2: Pyruvate kinase M2.

Cancer cells require sufficient energy and biosynthetic precursors for rapid proliferation. Unlike in normal cells, active glycolysis occurs in cancer cells even though oxygen is sufficient, which requires abundant glucose [3,4]. Pyruvate kinase (PK), a key rate-limiting enzyme in the Warburg effect, consists of four subtypes (PKM1, PKM2, PKL, and PKR) encoded by two groups of genes. PKM2 in cancer cells has become a key target for cancer treatment. Unlike other pyruvate kinases, PKM2 has tetrameric and dimeric forms (Figure 1). Tetramers, mainly PKM1, function as pyruvate kinase and regulate glycolysis, while dimers act as switches in energy metabolism and biosynthesis, converting the glucose metabolism of producing pyruvate [5]. Dimeric PKM2, as a transcription factor, translocates to the nucleus and activates and regulates the transcription of certain genes, including *β-catenin*. In addition, c-Myc, glucose transporter 1 (GLUT1), and lactate exporter monocarboxylate transporter 4 (MCT4), as the downstream protein of β-catenin, can be regulated [6,7]; therefore, PKM2-overexpression leads to increased glycolysis by increasing glucose input and lactate acid output, which speeds cancer progression [8,9]. In addition, the ratio of PKM2 determines whether the intracellular glucose metabolism is involved in the biosynthesis for proliferation or oxidative phosphorylation (OXPHOS) in the mitochondria [10].

Endogenous and exogenous activators and inhibitors can regulate the transition between the dimers and tetramers of PKM2. Tetramers have a seesaw-like pattern with allosteric regulatory domains, and they switch between the inactive T-state and active R-state [11]. Yuan et al. found that amino acids bind to an allosteric pocket (existing in PKM2 and PKM1); most of them stabilize PKM2 in an inactive T-state, and phenylalanine and alanine are almost 100% inhibited [12]. In addition, most amino acids show specificity for PKM2, with phenylalanine, arginine, glycine, and lysine showing a slight inhibitory effect (approximately 10%) on PKM1 [12,13]. Recently, novel naphthoquinone derivatives have been synthesized as selective inhibitors of PKM2, including compound **3K**. The anticancer and PKM2 inhibitory effects of compound **3K** were evaluated in vivo and in vitro. Currently, a novel 2, 3-didithiocarbamate-substituted naphthoquinone called compound **3h** (half-maximal inhibitory concentration [IC_50_] = 0.96 ± 0.18 μM), which is synthesized based on compound **3K**, provides potent inhibition of PKM2 that is superior to compound **3K** (IC_50_ = 2.95 ± 0.53 μM) [14].

Activation of the protein kinase B (Akt)/mechanistic or mammalian target of rapamycin (mTOR) plays a vital role in regulating cell survival and suppressing autophagy in cancer cells [15,16]. It has been proven that autophagy in human prostate cancer cells is induced by PKM2 knockdown via the Akt/mTOR pathway [17], and a specific PKM2 inhibitor, compound **3K**, also promoted the cell death of SK-OV-3 via restraining the Akt/mTOR pathway [18].

Although inhibiting PKM2 and glycolysis has been explored as an effective approach for cancer therapy, screening for novel PKM2 inhibitors and comparing their anticancer effects, thereby clarifying their action mechanisms, have not yet been reported; therefore, we found a specific PKM2 inhibitor called compound **3h**, evaluated its anticancer effects on prostate cancer cells, and performed molecular docking to further understand the mechanism of PKM2 inhibition in this study. Our results suggest that compound **3h** can suppress glycolysis by selectively inhibiting PKM2 expression and activity and then playing an anticancer role, which may provide a strategy for prostate cancer treatment.

## 2. Materials and Methods

### 2.1. Reagents

**Figure 2:**
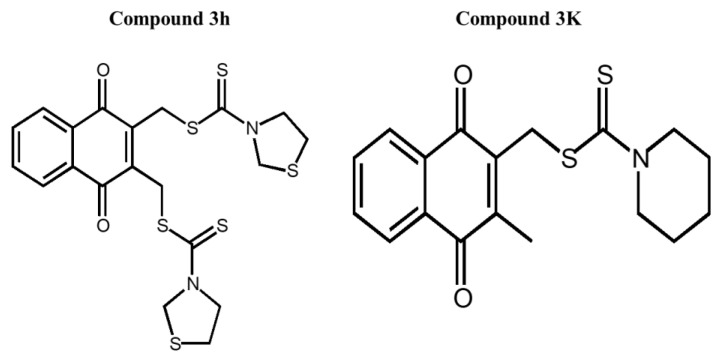
Chemical structures of compounds **3h** and **3K**.

Compounds **3h** and **3K** were dissolved in dimethyl sulfoxide (DMSO) (Sigma-Aldrich, St. Louis, MO, USA). Compound **3h** was synthesized by Professor In Su Kim (Department of Pharmacy, Sungkyunkwan University, Suwon, Republic of Korea) according to Ning’s method [14]. Compound **3K** (Catalog No. S8616) was purchased from SelleckChem (Houston, TX, USA). The structures of compounds **3h** and **3K** are shown in Figure 2. Roswell Park Memorial Institute (RPMI) 1640 medium (LM011-01) and fetal bovine serum (FBS) were purchased from Gibco Invitrogen Corporation (Carlsbad, CA, USA). Compound **3h** was diluted to an appropriate concentration in RPMI 1640 medium containing 10% FBS (S101-07, Welgene, Gyeongsan-si, Republic of Korea). Propidium iodide (PI) solutions and 4,6-diamidino-2-phenylindole (DAPI) were purchased from Sigma-Aldrich (St. Louis, MO, USA).

### 2.2. Antibodies

Primary antibodies against PKM2 (#4053S), PKM1 (#7067), poly (ADP-ribose) polymerase (PARP) (#9542), cleaved-PARP (#5625S), pro-caspase-7 (#9492), pro-caspase-3 (#9662), Cyclin-B1 (#4138), cell division cycle protein 2 homolog (Cdc2) (#77055), p-Cdc2 (#9111S), AMP-activated protein kinase (AMPK)-α (#2532), p-AMPKα (#2535), phosphatase and tensin homolog (PTEN) (#9559S), p-PTEN (#9551), Akt (#9272), p-Akt (#9271S), p-mTOR (#2971S), mTOR (#2972), ribosomal protein S6 kinase beta-1 (p70S6K) (#9202S), and p-p70S6K (#9206S) were purchased from Cell Signaling (Beverly, MA, USA). Primary antibodies against B-cell lymphoma 2 (Bcl-2) (sc-783), c-Myc (sc-40), Bcl-2-associated X (Bax) (sc-7480), GLUT1 (sc-7903), MCT4 (sc-376465), and β-actin (sc-47778) were purchased from Santa Cruz Biotechnology (Santa Cruz, CA, USA). Purchased primary antibodies against LC3B (ab51520), p62 (ab56416), and β-catenin (ab6302) were from Abcam (Abcam, Cambridge, UK). Beclin-1 (NB500-249) was purchased from Novus Biologicals (Littleton, CO, USA). Horseradish peroxidase (HRP)-conjugated secondary antibodies were purchased from Santa Cruz Biotechnology (Santa Cruz, Dallas, TX, USA).

### 2.3. Cell Lines and Cell Culture

All three human prostate cancer cell lines (LNCaP, PC3, and DU145) were purchased from the American Type Culture Collection (Manassas, VA, USA). Cultured cells in RPMI 1640 medium were supplemented with 10% heat-inactivated FBS, antibiotic–antimycotic (15240062) in a humidified, 5% CO_2_ atmosphere at 37 °C.

### 2.4. PK Assay

A PK assay kit (ab83432, Abcam, Cambridge, UK) was used to detect PK assay activity in LNCaP cells. In short, according to the guide, protein was extracted from cells (50 × 10^4^), pellets with four volumes of assay buffer and protein were added to a 96-well plate, then the PK assay buffer was added for a volume of 50 μL/well. The prepared reaction was mixed with the assay buffer, substrate mix, enzyme mix, and OxiRed™ probe provided in the PK assay kit, and the 50 μL/well reaction mix was added to the 96-well plate containing the protein. The standard linear curve was determined by serial dilutions of 1 nmol/mL standard pyruvate solution. After the reaction, the absorbance of all standards and samples was determined at a 570 nm wavelength using a VERSA Max Microplate Reader (Molecular Devices Corp., Sunnyvale, CA, USA).

### 2.5. Prepare Ligand and Receptor for Docking

All docking modeling procedures were performed using the Tripos Sybyl-X 2.1.1 (Tripos Inc., St Louis, MO, USA) molecular modeling package with a Windows 7 professional K operating system. First, compounds **3h** and **3K** were prepared with the sketch module and saved as mol2 format; we then assigned all atoms Gasteiger-Hückel charges. Before performing molecular docking, in order to obtain a stable conformation that converged to the maximum derivatives of 0.001 kcal mol−1.Å−1, we performed an energy minimization of ligands. The crystal structure of PKM2 in complex with phenylalanine (Protein Data Bank [PDB]:4FXJ) used in the experiment was downloaded from PDB. To prepare the receptor, the ligand phenylalanine was extracted, and all water molecules were deleted from the complex structure. The structure was then prepared with the protein preparation module of Sybyl-X 2.1.1 using the default parameters.

### 2.6. Molecular Docking of PKM2 and Compound **3K** and **3h**

In this study, we used Surflex-Dock embedded in Tripos Sybyl-X 2.1.1 to perform flexible docking. An SFXC file was constructed using a mol2 prepared protein structure. Phenylalanine was separated from the co-crystal structure of PKM2 (PDB: 4FXJ) to generate the binding site, and the binding site was saved as a protomol. The main setting was 20 solutions per compound, and other parameters accepted the Surflex-Dock Geom settings. Surflex-Dock scoring function was used to calculate the binding affinity (−log Kd) for each docking model. Subsequently, the consensus scores were calculated to compare the Total Score, PMF score, ChemScore, G score, and D score. Lastly, we selected the best docking model equipped with excellent binding affinity, consensus scores (≥3), and the number of intermolecular interactions.

### 2.7. Cytotoxicity Assay

LNCaP, PC3, and DU145 cells (1 × 10^4^ or 5 × 10^3^ cells) were seeded into a 96-well plate and after 24 h incubation, different concentrations of compounds **3h** and **3K** were made and then treated for 24 or 48 h. A total of 10 μL/well EZ-Cytox cell viability (WST) assay kit (EZ-BULK150, Daeillab, Republic of Korea) was added and incubated, followed by detecting the absorbance measurement at 450 nm with a VERSA Max Microplate Reader (Molecular Devices Corp., CA, USA) to determine cell viability. Finally, according to absorbance measurement, we calculated the IC_50_ using Sigma Plot 10.0 software.

### 2.8. Proliferation Assay

Plated LNCaP (5 × 10^3^ cells/well) were placed into a 96-well plate and incubated until the confluency of cells achieved 50%. Then, we treated compound **3h** (0, 2.5, 5, and 10 µM) and put the 96-well plate into IncuCyte® ZOOM 2016B software (Essen Bioscience, Ann Arbor, MI, USA) to acquire the images every **3h**, and analyze the confluency.

### 2.9. Colony Forming Assay of LNCaP cells

A total of 1000 cells/well was seeded into a 6-well plate. It was treated with either compound **3h** or control for 24 h after 5 days incubation, and then the cells were fixed and stained overnight with 4% paraformaldehyde (J19943, Thermo Fisher Scientific, Waltham, MA, USA) and 0.5% crystal violet (CR1035-100-00, Biosesang, Seongnam, Republic of Korea). ImageJ software 1.52a (NIH, Bethesda, MD, USA) was used to analyze the relative colony area.

### 2.10. Cell Cycle Analysis

LNCaP cells (30 × 10^4^ cells/dish) were seeded into 100-π dishes for 24 h incubation. Amounts of 0, 2.5, 5, and 10 μM compound **3h** were treated to LNCaP for 48 h, the attached cells were harvested and put into 70% cold ethyl alcohol (EtOH) to fix overnight at 4 °C. Then, cells were resuspended in phosphate-buffered saline (PBS), and stained with a mixed solution of 0.5 mL PBS and 5 μL of PI (P4864-10ML, Sigma-Aldrich, St. Louis, MO, USA) and 10 mg/mL RNase A. In the last step, we measured the effect of compound **3h** on the cell cycle in LNCaP cells using a NovoCyte flow cytometer (ACEA Biosciences, Santa Clara, CA, USA).

### 2.11. Western Blot Analysis

LNCaP cells were seeded at a density of 80 × 10^4^ cells/dish in 100-π dishes and compound **3h** (0, 2.5, 5, and 10 μM) was treated afterwards. Cells were harvested and then lysed with PRO-PREP^TM^ extraction solution (17081, iNtRON, Seongnam-shi, Republic of Korea) on ice for 30 min. Before Western blotting, we conducted a bicinchoninic acid (BCA) assay (23228, Thermo Fisher Scientific, Waltham, MA, USA) to measure protein concentrations in the different samples according to the manufacturer’s instructions. After electrophoresing 30 μg protein on 6–15% sodium dodecyl sulfate polyacrylamide gels for 30 min under 80 V, and for 1 h 30 min under 100 V, we transferred the protein to olyvinylidene fluoride (PVDF) membranes (Millipore, Billerica, MA, USA) for 1 h 10 min under 400 mA. We followed this by blocking with skimmed milk for 1 h, and then incubated the PVDF membranes with all kinds of primary antibodies for at least 8 h at 4 °C. The following day, horseradish peroxidase-conjugated anti-rabbit IgG (NB7176, 1:20000; Novus Biologicals, Littleton, CO, USA) or anti-mouse IgG (NB7561, 1:40000; Novus Biologicals, Littleton, CO, USA) secondary antibodies were incubated with PVDF membranes for 1 h at room temperature. At the last step, the Immobilon Forte Western HRP (Merck Millipore, Burlington, MA, USA) and WSE-6200 LuminoGraph Ⅱ Imaging System (ATTO, Tokyo, Japan) were used to visualize and detect the protein bands. The intensities of protein bands were quantified with ImageJ software 1.52a (NIH, Bethesda, MD, USA).

### 2.12. Apoptosis

After treatment for 48 h with compound **3h**, LNCaP cells were harvested and stained with 2 μL Annexin V-FITC (556419, BD Biosciences, San Diego, CA, USA) and 4 μL PI in 100 μL of 1 × binding buffer for 30 min. After diluting cells with 1 × binding buffer, an apoptosis assay was performed using a NovoCyte flow cytometer (ACEA Biosciences, Santa Clara, CA, USA).

### 2.13. DAPI Nuclear Staining

Apoptosis can be identified based on the presence of broken, condensed, degenerated nuclei, which can be stained by DAPI. LNCaP cells (5 × 10^4^) seeded in confocal dishes were treated with DMSO or compound **3h** (2.5, 5, and 10 µM) for 48 h. Acetone was used for fixation, and 0.1 μg/mL DAPI (D9542, Sigma-Aldrich, St. Louis, MO, USA) was used to stain DNA. At the last step, a confocal K1-fluo microscope (Nanoscope Systems, Daejeon, Republic of Korea) was used to observe morphological changes in the nucleus.

### 2.14. Stain LNCaP Cells with Acridine Orange

Seeded LNCaP cells (5 × 10^4^) in confocal dishes were treated with DMSO or compound **3h** (2.5, 5, and 10 µM) for an extra 48 h. LNCaP was stained with 1 μg/mL acridine orange (00910250, Thermo Fisher Scientific, Waltham, MA, USA) for 15 min. Last, stained cells were observed under a Confocal K1-fluo microscope (Nanoscope Systems, Daejeon, Republic of Korea) at 400×/600× magnification.

### 2.15. Seahorse XFe96 Analysis of Cell Mito Stress Test and Glycolytic Rate Assay

The Agilent Seahorse Xfe cell mito stress test (Kit 103015-100) evaluated key parameters of mitochondrial function by directly measuring the OCR, and after mitochondrial inhibition, a glycolytic rate assay (Kit 103344-100) accurately measured glycolytic rates under basal glycolysis and compensatory glycolysis. During both experiments, the OCR and ECAR were continuously monitored and recorded by a Seahorse Xfe96 analyzer (Agilent, Santa Clara, CA, USA). Briefly, on the day before detecting with the Seahorse Xfe96 analyzer, LNCaP cells seeded into a Xfe96 cell culture microplate were treated with DMSO or compound **3h** (2.5, 5, and 10 µM), and calibrated by an XFe cartridge with 200 μL XF calibration buffer overnight. On the day of assay, according to the user guide, XF assay medium was used to wash cells; different compounds (oligomycin, carbonyl cyanide-*p*-trifluoromethoxyphenylhydrazone (FCCP), Rotenone and antimycin A (ROT/AA) for the cell mito stress test, and ROT/AA, 2-Deoxy-D-glucose (2-DG) for the glycolytic rate assay) were added to the injection ports for the cell mito stress test and glycolytic rate assay. After detection, raw data were analyzed automatically by Assay Generator.

### 2.16. Statistical Analysis

All data are expressed as the mean ± standard error (SEM) of at least three independent experiments. Our analysis was conducted using GraphPad Prism software 5.0 (GraphPad Software, San Diego, CA, USA). Statistical significance was analyzed using one-way analysis of variance followed by Bonferroni’s post hoc comparisons test. Statistical significance was set at *p* value < 0.05.

## 3. Results

### 3.1. Anti-Proliferation Effects of Compound **3h** in Three Prostate Cancer Cell Lines

**Figure 3:**
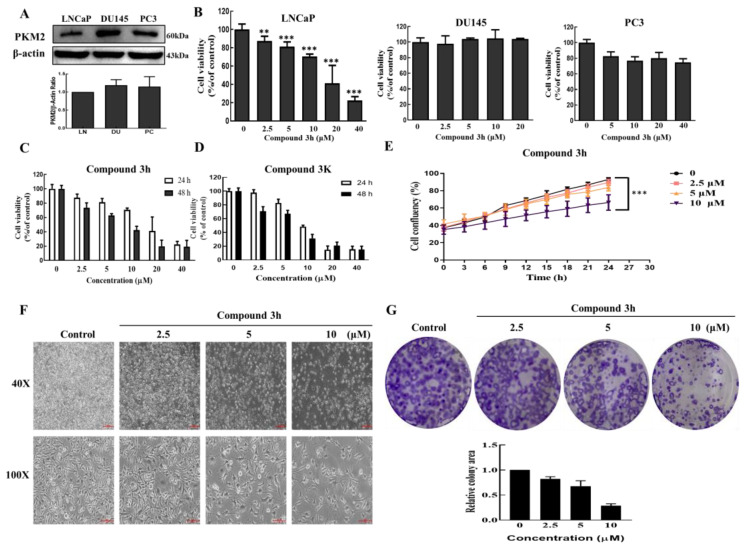
Cytotoxicity of compounds **3h** and **3K** on three different prostate cancer cell lines. (**A**) The expression of PKM2 LNCaP, DU145, and PC3. (**B**) Cell viability of compound **3h**. Compound **3h** was treated in LNCaP, DU145, or PC3 cells for 24 h. (**C**) Anticancer effect of compound **3h** on LNCaP cells. Cell viability of LNCaP decreased with the increase in compound **3h** concentration (0, 2.5, 5, 10, 20, and 40 μM) for 24 and 48 h treatment. Cell viability was assessed using WST assay kit. (**D**) Cytotoxicity effect of compound **3K** on LNCaP cells for 24 h and 48 h. (**E**) Compound **3h** suppressed the proliferation of LNCaP cells. (**F**) Compound **3h** treatment for 48 h caused morphological changes in LNCaP cells. Scale bar, 200 μm (40×), 100 μm (100×). (**G**) Compound **3h** treatment inhibited colony formation in LNCaP and the quantitative analysis. The values represent the mean ± SD. ** *p* < 0.01 and *** *p* < 0.001. The uncropped blots are shown Supplementary Material.

To evaluate the anticancer effects of compound **3h** in three different human prostate cancer cell lines, the in vitro cytotoxicity of compound **3h** was assessed in LNCaP, DU145, PC3 cells. WST results indicated that compound **3h** had a higher inhibitory effect on LNCaP cells than on the other two cell lines, even though DU145 and PC3 cells had higher protein expressions of PKM2 (Figure 3A,B). Since compound **3K** has been proven to be a specific PKM2 inhibitor and shows anticancer effects in various human cancer lines, including SK-OV-3, HCT116, and Hela [18,19], this study compared the anticancer effects of compound **3h** and compound **3K** on the LNCaP cell line. WST results indicated that both compounds had similar inhibitory effects on LNCaP after 48 h of treatment (Figure 3C,D). The results of the IncuCyte Zoom assay proved that compound **3h** inhibited the growth of LNCaP in a concentration-dependent manner (Figure 3E). The effect of compound **3h** on LNCaP cell morphology can be seen in Figure 3F. As shown in Figure 3G, colony formation was significantly inhibited after compound **3h** treatment.

### 3.2. PKM2 Expression Was Selectively Inhibited by Compound **3h**, Rather Than PKM1

**Figure 4:**
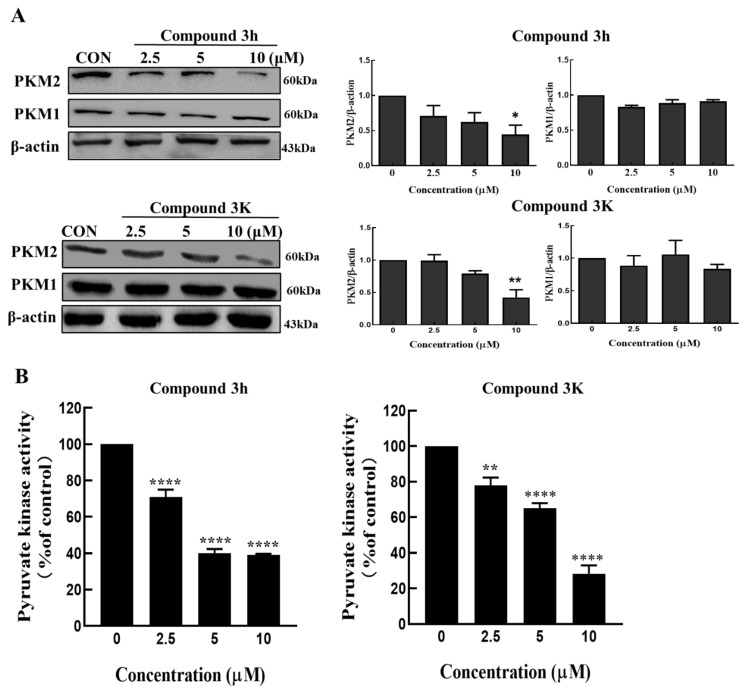
Compound **3K** and **3h** selectively inhibit PKM2 rather than PKM1. (**A**) Compounds **3K** and **3h** have a specific selectivity in reducing PKM2 expression in LNCaP cells. (**B**) The activity of pyruvate kinase in LNCaP cells was inhibited by compound **3h** and compound **3K** depending on concentration. The values represent the mean ± SD. * *p* < 0.05, ** *p* < 0.01, and **** *p* < 0.0001. The uncropped blots are shown Supplementary Material.

Both compound **3h** (IC_50_ = 0.96 ± 0.18 μM) and compound **3K** (IC_50_ = 2.95 ± 0.53 μM) showed high inhibitory activity against PKM2 [14]. To confirm how compounds **3h** and **3K** affected the expression of pyruvate kinase in LNCaP cells, we performed Western blotting with antibodies against PKM1 and PKM2. As shown in Figure 4A, from decreased PKM2 expression, compounds **3h** and **3K** had a specific selectivity for PKM2 rather than PKM1 in LNCaP cells. To confirm the effects of compounds **3h** and **3K** on the pyruvate kinase activity in LNCaP cells, we treated LNCaP cells with different concentrations of compounds **3h** and **3K**; both were able to impair the pyruvate kinase activity in a concentration-dependent manner (Figure 4B). In conclusion, compound **3h** inhibited selectively the pyruvate kinase activity and expression of PKM2, but not PKM1 in LNCaP cells, similar to compound **3K**.

### 3.3. Molecular Docking Analysis of Compounds **3h** and **3K**

**Figure 5:**
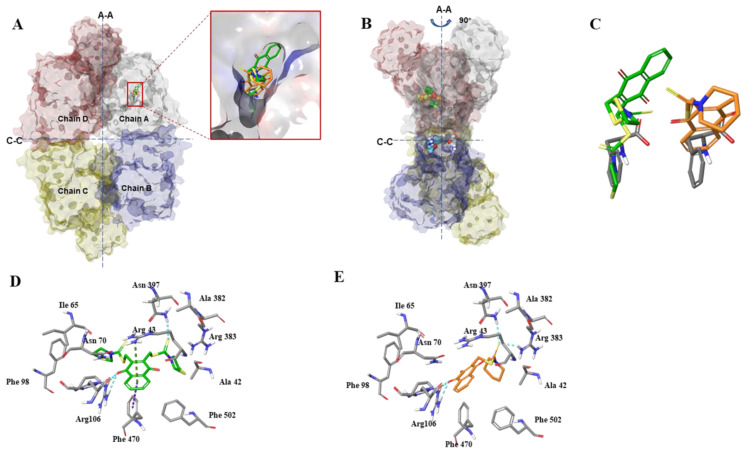
Binding mode of compounds **3h** and **3K** in the effector phenylalanine-binding site of PKM2. (**A**) Superimposition and enlarged view of phenylalanine (gray sticks), compound **3h** (green sticks), and compound **3K** (faded orange sticks) over the X-ray of PKM2. PKM2 tetramer is shown with the effector site complexed with the original ligand phenylalanine (gray sticks), compound **3h** (green sticks) and compound **3K** (faded orange sticks). The chain is colored, light gray is for Chain A, cyan Chain is for B, green is for Chain C, magenta is for Chain D. The large (A-A) and small (C-C) interfaces between monomers are shown as dashed lines. (**B**) Side view (rotated 90° to that of A) of PKM2 tetramer complexed with the original ligand phenylalanine (gray sticks), and compound **3h** (green sticks) and compound **3K** (faded orange sticks). (**C**) Superimpositions of phenylalanine (gray) and compound **3h** (green) or **3K** (faded orange). (**D**,**E**) Close-up view of the key interactions involved in bonding with compound **3h** (**D**, green sticks) and compound **3K** (**E**, faded orange sticks) in effector-binding pocket. Key residues surrounding compound **3h** and **3K** are shown as gray sticks and labeled. π-π stacking is depicted by dotted purple lines. π-cation is shown as dotted dark green lines. Hydrogen bonds are depicted as dotted cyan lines.

To elucidate the molecular binding basis of the interaction between both compounds and PKM2, we investigated the possible binding modes of compounds **3h** and **3K** by docking stimulation using Surflex-Dock. The allosteric binding of small-molecule metabolites can enable PKM2 to exist in different isoforms, with equilibrium between monomers and tetramers. Among the metabolites, phenylalanine works as a PKM2 inhibitor that stabilizes it in an inactive T-state tetrameric conformer. Thus, the crystal structure of PKM2 complexed with phenylalanine (PDB ID: 4FXJ), that remains in an inactive T-state, was selected as the target receptor to carry out the docking stimulation of compounds **3K** and **3h** [12]. According to our docking results in Figure 5A–C, the binding modes of compounds **3h** and **3K** were almost identical and similar to the X-ray of phenylalanine, which was well-occupied in the allosteric pocket, further stabilizing PKM2 in the inactive T-state. X-ray structure analysis revealed a polar interaction network in the binding pocket of phenylalanine (carboxylate group of phenylalanine with Asn70 and Arg106), which is crucial for PKM2 inhibition activity. Likewise, in the docked models of PKM2 with **3h** and **3K** (Figure 5D,E), the carboxylate moiety of **3h** also formed hydrogen bonds with Arg106, and **3K** formed hydrogen bonds with Arg106. In addition, the S atom of the carbonyl sulfide of **3K** interacted with Asn397 and Arg383 by forming an H-bond, whereas **3h** only formed hydrogen bonds with Asn397. We also identified unique T-shaped π-π and cation-π interactions between the phenyl ring of **3h** and Phe470 and Arg43, respectively, in the backbone of PKM2. Thus, these two unique interactions might contribute a higher binding affinity for the inhibitory effect of PKM2, consistent with the docking score (−log Kd) of **3h** (5.6815) being higher than that of **3K** (5.1002).

### 3.4. Compound **3h** Induces Apoptotic Cell Death in LNCaP Cells

**Figure 6:**
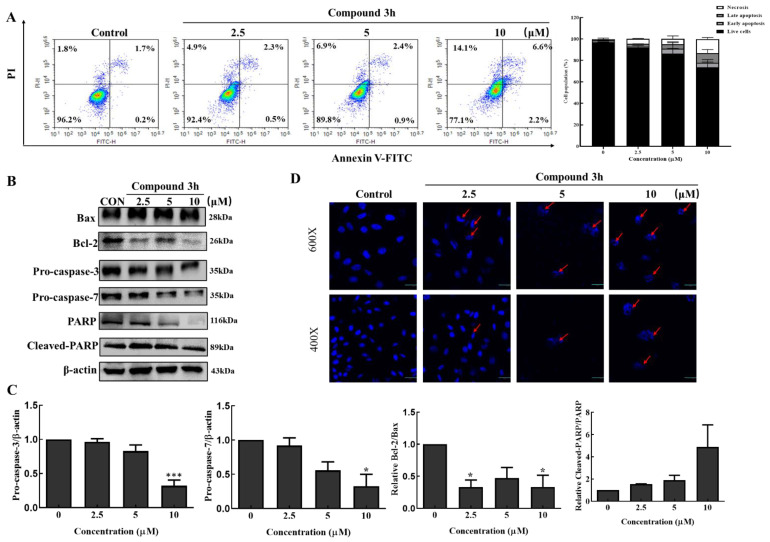
Compound **3h** induces LNCaP cellular apoptotic cell death. (**A**) Compound **3h** caused apoptosis in LNCaP cells. Late apoptosis and necrosis percentage increased in the compound **3h**-treated group. (**B**) Effect of compound **3h** on the expression levels of apoptotic proteins. Compound **3h** induced apoptotic proteins expression. (**C**) Quantitative data of apoptotic proteins. (**D**) Nuclear morphological changes and apoptotic bodies were detected in the compound **3h**-treated group. The values represent the mean ± SD. * *p* < 0.05, and *** *p* < 0.001. The uncropped blots are shown Supplementary Material.

Here, we elucidated the mechanisms involved in the anticancer activity of compound **3h** after treatment on LNCaP. As shown in Figure 6A, the percentage of late apoptosis increased in the compound **3h**-treated group by 2.3% (with 2.5 μM), 2.4% (with 5 μM), and 6.6% (with 10 μM) compared with 1.7% in the control group. Similarly, the necrosis percentage increased depending on concentration (1.8% in the control, 4.9% in the 2.5 μM, 6.9% in the 5 μM, and 14.1% in the 10 μM groups). As shown in Figure 6B, Bax and cleaved-PARP increased, whereas Bcl-2, pro-caspase-3, pro-caspase-7, and PARP decreased. The relative Bcl-2/Bax ratio decreased, and the relative cleaved-PARP/PARP ratio markedly increased (Figure 6C). DAPI staining was carried out to examine the nuclear morphology and apoptotic bodies of apoptosis via confocal microscopy, which were observed in the compound **3h**-treated group (Figure 6D).

### 3.5. Compound **3h** Slightly Induces LNCaP Cells Arrest at G2/M Phase

**Figure 7:**
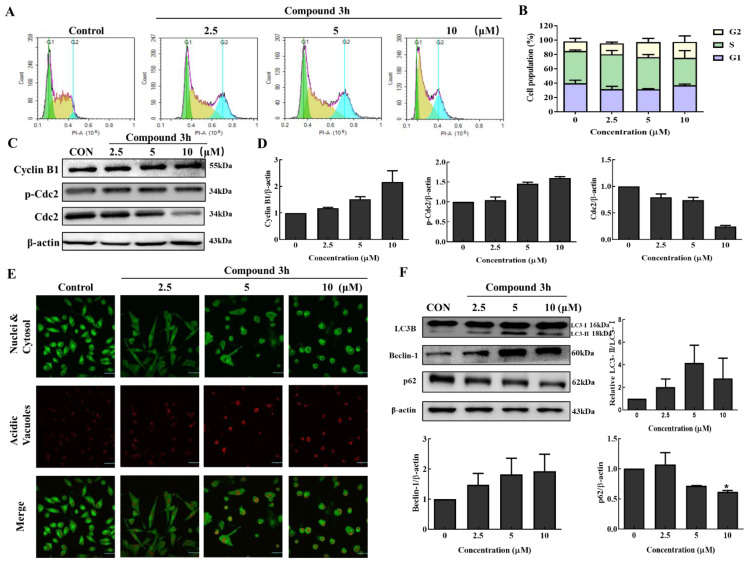
Compound **3h** induces G2/M arrest and autophagy in LNCaP cells. (**A**) Cell cycle results of LNCaP measured by NovoCyte after treating compound **3h** (0, 2.5, 5, and 10 μM). (**B**) Bar graph indicates the cell population involved in G1, S, and G2/M phases in LNCaP cells after compound **3h** treatment. (**C**) Expression changes in G2/M arrest-related proteins (Cyclin B1, p-Cdc2, Cdc2) in LNCaP cells after compound **3h** treatment. (**D**) Representative histogram showing the expression levels of Cyclin B1, p-Cdc2, Cdc2, compared to β-actin in LNCaP. (**E**) Autophagy activation was clearly observed in LNCaP cells after compound **3h** treatment. Red fluorescence intensity of autophagic vacuoles was observed in compound **3h**-treated group in a concentration-dependent manner (Magnification 600×). (**F**) Expression of autophagic proteins in LNCaP cells after compound **3h** treatment and LC3-Ⅱ/LC3-Ⅰ, relative Beclin-1, and relative p62 were calculated and shown as graphs. The values represent the mean ± SD. * *p* < 0.05. The uncropped blots are shown Supplementary Material.

PKM2 inhibition or PKM2 knockdown is associated with G2/M arrest in various cancer cell lines, including DU145 and SK-O-V3 [17,18]. To explore whether compound **3h** induced G2/M arrest in LNCaP, we analyzed DNA content using a NovoCyte flow cytometer after compound **3h** treatment for 48 h. According to the relative distribution of cells in Figure 7, G2/M arrest was slightly induced (Figure 7A,B), cell cycle-related proteins, p-Cdc2 and Cyclin B1 were upregulated, and Cdc2 was markedly downregulated (Figure 7C,D).

### 3.6. Compound **3h** Induces Autophagic Cell Death in LNCaP Cells

To analyze whether autophagy was induced in the compound **3h**-treated group, the acridine orange staining assay was performed. The emission color of acridine orange changed from yellow to orange to red as the pH dropped in the acidic vacuole upon autophagy. Autophagic vacuoles with red fluorescence intensity were observed in the compound **3h**-treated group in a concentration-dependent manner (Figure 7E). LC3-Ⅱ is the autophagic vacuole-related form, so the transformation of LC3-Ⅰ to LC3-Ⅱ is an important step in autophagosome formation. Beclin-1 and LC3-Ⅱ levels increased, while p62 decreased in the cells (Figure 7F).

### 3.7. Compound **3h** Suppresses the Akt/AMPK/mTOR Pathway in LNCaP

**Figure 8:**
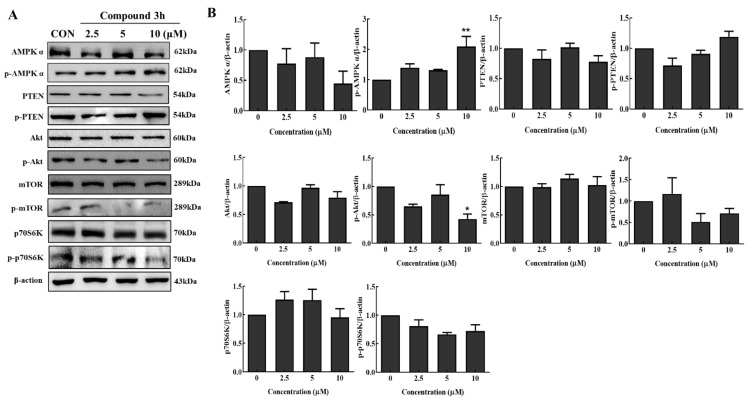
Changes in protein expression in signaling pathways in LNCaP cells after PKM2 inhibition by compound **3h**. (**A**) Expression levels of proteins related to Akt/AMPK/mTOR signaling pathway. Compound **3h** treatment suppressed the activation of Akt/mTOR. (**B**) Representative histogram showing the expression levels of proteins. The values represent the mean ± SD. * *p* < 0.05, and ** *p* < 0.01. The uncropped blots are shown Supplementary Material.

Akt/AMPK/mTOR has been studied as a typical anticancer pathway involved in autophagy. To further understand the mechanism involved in the anticancer effect of compound **3h**, according to our results in Figure 8A,B, the Akt/mTOR pathway was suppressed by compound **3h** treatment in LNCaP. In addition, as we can see, after compound **3h** treatment, the expression of p-PTEN increased and p-Akt decreased, which restrained mTOR phosphorylation; thereby, autophagy was promoted. Although the expression levels of total Akt and mTOR were similar between the experimental and control groups, increased p-AMPKα further inhibited mTOR phosphorylation. Increased AMPK phosphorylation curbed mTOR aivity and downregulated protein synthesis to inhibit cell proliferation. Therefore, p70S6K and p-p70S6K, as the main targets of mTOR, were analyzed. p-p70S6K levels decreased in response to decreased p-mTOR, and increased p-AMPKα levels after PKM2 was inhibited. These results indicated that compound **3h** suppressed the prostate cancer cell line, LNCaP, by regulating the Akt/AMPK/mTOR pathway.

### 3.8. Mitochondrial Activity and Glycolysis in LNCaP Cells Were Impaired by Compound **3h**

**Figure 9:**
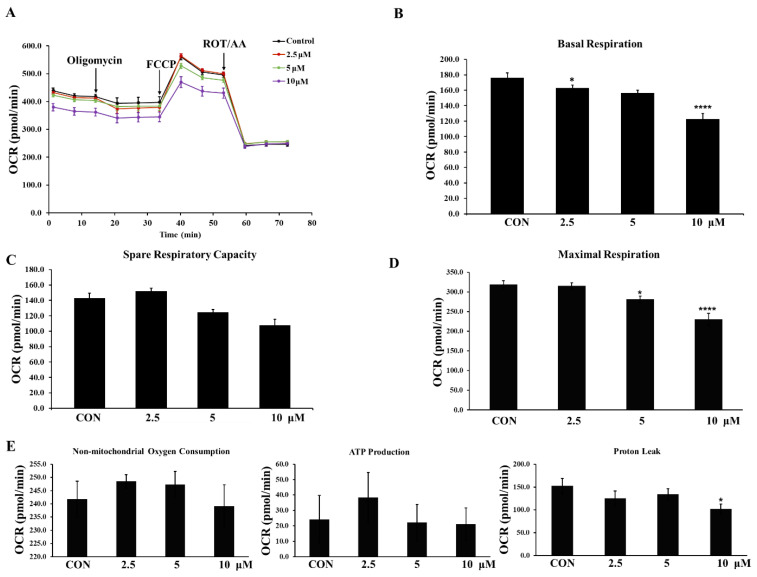
PKM2 specific inhibitor, compound **3h**, impairs the mitochondrial activity in LNCaP cells, leading to decreased basal respiration, spare respiratory capacity, and maximal respiration. (**A**) Profile of mito stress test data for oxygen consumption rate (OCR) (pmol/min) in LNCaP cells with arrows indicating injections into media of specific stressors. (**B**–**E**) Results of Seahorse XFe cell mito stress test generator report shown for OCR (pmol/min). B is for basal respiration, C is for spare respiration capacity, D is for maximal respiration, E is for non-mitochondrial oxygen consumption, ATP production, and proton leak. The values represent the mean ± SD. * *p* < 0.05 and **** *p* < 0.0001.

The Seahorse analyzer was used to analyze the bioenergetic changes in LNCaP cells after compound **3h** treatment. Cellular bioenergetic markers, including OCR and proton efflux rate (PER), were detected. As shown in Figure 9A, compound **3h** inhibited OXPHOS, as indicated by the decreased OCR. Decreased basal respiration, spare respiratory capacity, and maximal respiration indicated that compound **3h** regulated the mitochondrial dynamics (Figure 9B–E).

**Figure 10:**
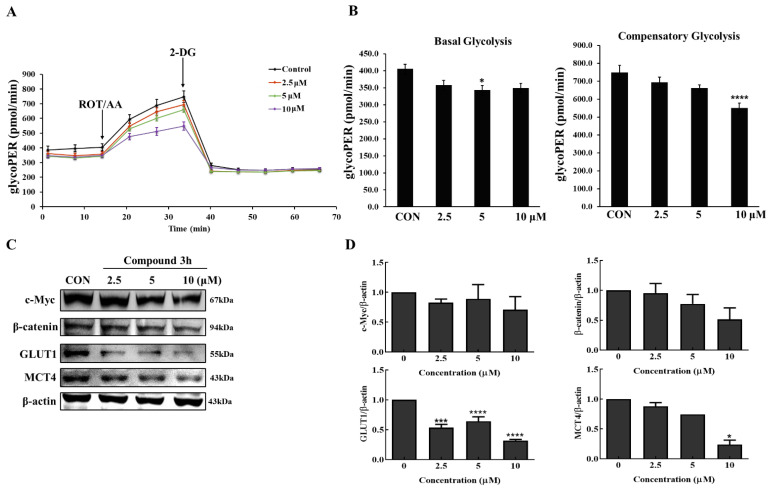
Compound **3h** impairs the glycolytic function in LNCaP cells. (**A**) Profile of glycolytic rate assay in LNCaP with specific injections into media. (**B**) Results of Seahorse XF glycolytic rate assay-generator report for basal and compensatory glycolysis (pmol/min). (**C**) Protein expression levels of c-Myc, β-catenin, GLUT1, MCT4 were evaluated by Western blot after compound **3h** treatment. (**D**) Representative histograms showing the expression levels of c-Myc, β-catenin, GLUT1, MCT4. The values represent the mean ± SD. * *p* < 0.05, *** *p* < 0.001. and **** *p* < 0.0001. The uncropped blots are shown Supplementary Material.

The compound **3h**-treated group showed decreased basal glycolysis and compensatory glycolysis after measuring glycoPER (Figure 10A,B). Dimer PKM2 translocated into the nucleus to perform non-metabolic functions, where β-catenin expression was unregulated, thereby increasing the expression of c-Myc and GLUT1. As shown in Figure 10C,D, compound **3h** not only decreased β-catenin and c-Myc expression levels, but it also significantly decreased the expression levels of GLUT1 and MCT4, thereby decreasing the rates of glucose uptake and lactate efflux, respectively. Therefore, compound **3h** decreased the glycolytic activity in LNCaP cells by downregulating β-catenin and c-Myc levels.

## 4. Discussion

Prostate cancer is a common malignant cancer characterized by slow progression, with a gradually increasing incidence, and a high mortality rate in advanced cases. Although the 5-year survival rate of localized prostate cancer is >99%, advanced prostate cancer is regarded as incurable [20,21]. Although androgen deprivation therapy (ADT) is considered to be the primary treatment option for advanced prostate cancer, most patients develop recurrence and insensitivity after an initial positive response [22]. Docetaxel significantly improves the overall survival of castration-resistant prostate cancer (CRPC) patients, and has been approved as a first-line treatment option for CRPC [23]. However, many patients develop resistance to docetaxel. Therefore, the development of novel agents as new targets for prostate cancer treatment is essential to solve the problems associated with chemotherapy.

Currently, the Warburg effect, as a hallmark of cancer, may be a breakthrough for cancer therapy. PKM2 plays an important role in the regulation of anabolic metabolism, including the Warburg effect. High PKM2 expression was previously shown in prostate cancer tissues [24]; therefore, targeting PKM2 or glycolysis may be a potent strategy for prostate cancer treatment. Previously, it was shown that PKM2 knockdown in the PKM2-overexpressed prostate cancer cell line DU145 induced autophagic cell death by influencing cellular metabolism and the Akt/mTOR pathway [17]; however, PKM2 knockdown therapy is severely limited in clinical treatment. Therefore, exploring novel anticancer agents that target PKM2 remains a hotspot of research. Recently, the anticancer effect of several PKM2 inhibitors (shikonin, metformin) has been identified. In particular, the inhibitory effect of compound **3K**, a PKM2 specific inhibitor, on SK-OV-3 has been proven in vitro and in vivo [18]. In this study, we evaluated the anticancer effect of compound **3h**, which was synthesized based on the structure of compound **3K** and its molecular mechanism in LNCaP. Our results provide a new perspective for the development of novel agents for prostate cancer treatment. We found that compound **3h** treatment decreased the proliferation and colony formation of LNCaP cells in a dose- and time-dependent manner. According to a previous report, PKM2 knockdown was related to G2/M arrest in HeLa and SiHa cells [25]; therefore, we assessed the influence of compound **3h** on the cell cycle in LNCaP cells. Compound **3h** treatment led to a slight increase in the percentage of cells in the G2/M phase, which was mediated by increased p-Cdc2 expression.

Abundant glucose uptake and lactate elimination are essential features of aerobic glycolysis in cancer cells [4]. In this study, compound **3h** inhibited PKM2 expression in LNCaP cells, which led to the inhibition of aerobic glycolysis, reduction of glucose intake and lactate elimination—consistent with our results of downregulated GLUT1 and MCT4 expression—and decreased basal glycolysis and compensatory glycolysis. It has been reported that the mTOR-MFN2-PKM2 signaling axis couples glycolysis and mitochondrial OXPHOS, which increases the phosphorylation of MFN2, leading to increased PKM2:MFN2 interaction and OXPHOS increase [26]. Therefore, compound **3h** may impair OXPHOS and glycolysis by inhibiting PKM2 and interfering with the PKM2:MFN2 interaction.

PKM2 has dimeric and tetrameric forms. Dimeric PKM2 translocates to the nucleus and regulates transcription factors (including β-catenin), thus affecting a variety of signaling pathways that can promote tumor progression [27,28]. Tetrameric PKM2 has allosteric regulatory domains, and PKM2 can be stabilized in the inactive T-state when inhibitory phenylalanine binds to the binding pocket [12]. According to our molecular docking results, compound **3h** can bind to the phenylalanine-binding pocket at a similar position; therefore, compound **3h** also stabilizes PKM2 in the inactive T-state, which inhibits PKM2 activity and blocks dimer formation, causing inhibition of glycolysis and nuclear translocation of dimeric PKM2, further suppressing cancer cell proliferation.

Autophagic death is a common pathway of cell death. Activation of the Akt/mTOR pathway can inhibit autophagy, which is critical for cancer cell growth and survival [29]. Akt is a major regulator of cell survival under stressful conditions [30], which not only regulates nutrient intake, but also the expression of growth factors [31]. P70S6K is a downstream target of mTOR, and can be upregulated and phosphorylated by mTOR phosphorylation; thus, more downstream proteins associated with proliferation will be activated and induced [32]. In addition, AMPK phosphorylation induces mTOR inhibition, leading to autophagy inhibition [33]. It was reported that the PKM2 knockdown, and PKM2 inhibitor, compound **3K**, inhibited mTOR phosphorylation and autophagy activation by activating AMPK phosphorylation in DU145 cells and SK-OV-3 cells, respectively [17,18]. In this study, according to the Western blot results, compound **3h** increased the expression of p-AMPKα, and decreased that of PKM2, p-Akt, p-mTOR, and p-p70S6K; therefore, compound **3h** inhibited PKM2, which caused autophagic cell death by inhibiting the Akt/mTOR signaling pathway in LNCaP.

Apoptosis and autophagy are key cellular processes that maintain cellular homeostasis and are connected in complex ways. Bcl-2 and Beclin-1 play important roles in the connection between apoptosis and autophagy [34]. Bcl-2 activation can be modulated by post-translational modifications such as phosphorylation of Bad. Bad is phosphorylated by Akt and p70S6K, which inhibits its interaction with anti-apoptotic Bcl-2 proteins, and leads to the inhibition of Bax-triggered apoptosis [35]. Beclin-1 is a lipid-kinase complex that is involved in autophagosome nucleation. Beclin-1 conjugates with members of the Bcl-2 family through the BH3 domain, and dissociation from Bcl-2 is critical for its autophagic activity [36]. PKM2 knockdown activates apoptotic and autophagic signaling pathways in different cancer cells, including DU145 and A549 cells [17,37]. So, PKM2 knockdown and PKM2 inhibition influence the interaction between Bcl-2 and Beclin-1, which may be accompanied by a decrease in Bcl-2 expression, and a Beclin-1 dissociation. In this study, compound **3h** inhibited Akt and p70S6K phosphorylation, which may have promoted Bax-triggered apoptosis through the interaction between Bcl-2 and Bad. In addition, Bcl-2 decreased after compound **3h** treatment, and more Beclin-1 was dissociated, which led to autophagic death. Our results suggested that compound **3h** inhibited PKM2, and induced apoptosis and autophagy in LNCaP cells.

## 5. Conclusions

In conclusion, our study showed that compound **3h**, which acts as a specific PKM2 inhibitor, inhibited PKM2 activity and expression, and further significantly inhibited the glycolysis and proliferation of LNCaP cells. Compound **3h** decreased the nuclear translocation of dimeric PKM2, which decreased the expression of β-catenin, further decreasing the expression of GLUT1 and MCT4. Combined with the results of Western blotting, compound **3h** exerted anti-tumor effects in LNCaP cells by inhibiting glycolysis, and inducing apoptosis and autophagy mediated by the Akt/mTOR signaling pathway.

## Data Availability

Data and materials generated are relevant to the results that are included in this article. Other data are available from the corresponding author Kim upon reasonable request.

## Supplementary Materials

The following supporting information can be downloaded at: https://www.mdpi.com/article/10.3390/cancers15010265/s1, The uncropped blots are shown Supplementary Material.
