# Supplementary material for: Novel Specific Pyruvate Kinase M2 Inhibitor, Compound 3h, Induces Apoptosis and Autophagy through Suppressing Akt/mTOR Signaling Pathway in LNCaP Cells
Source: Cancers (Basel). 2022 Dec 30;15(1):265. doi: 10.3390/cancers15010265 (PMC9818605; doi:10.3390/cancers15010265)
Supplement: Supplementary file 1 [file cancers-15-00265-s001.zip › cancers-2113067-supplementary.pdf]

cancers

MDPI

Supplementary Material: Novel Specific Pyruvate Kinase M2 Inhibitor, Compound 3h, Induces Apoptosis and Autophagy through Suppressing Akt/mTOR Signaling Pathway in LNCaP Cells

Chunxue Jiang, Xiaodi Zhao, Taejoo Jeong, Ju Young Kang, Jae Hyeon Park, In Su Kim and Hyung Sik Kim

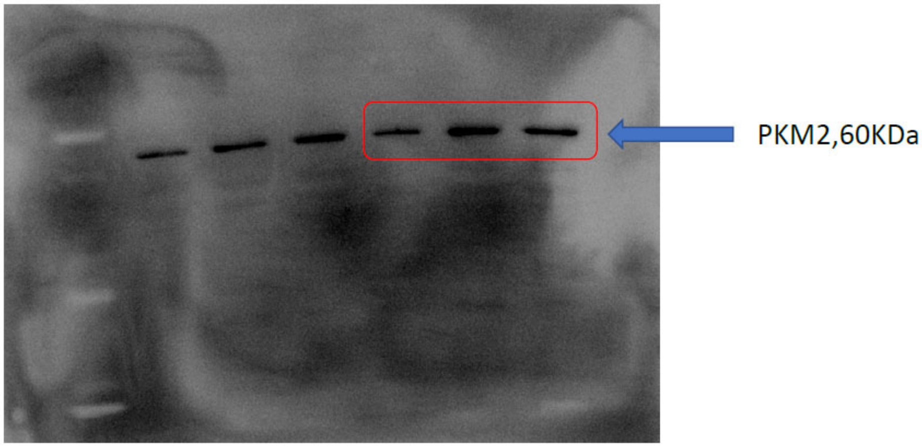

Figure S1. Raw data for Figure 3A.

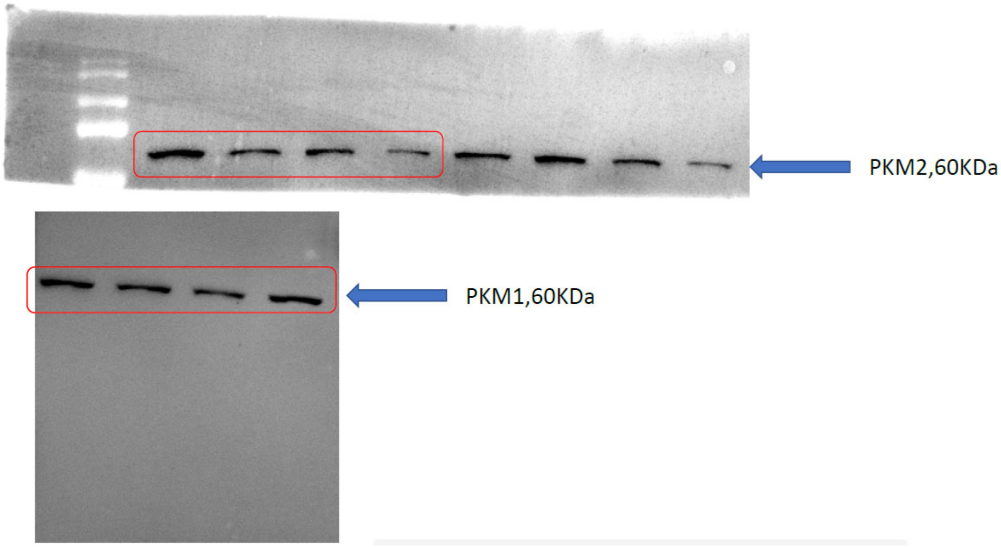

Figure S2. Raw data for Figure 4A.

Compound 3K

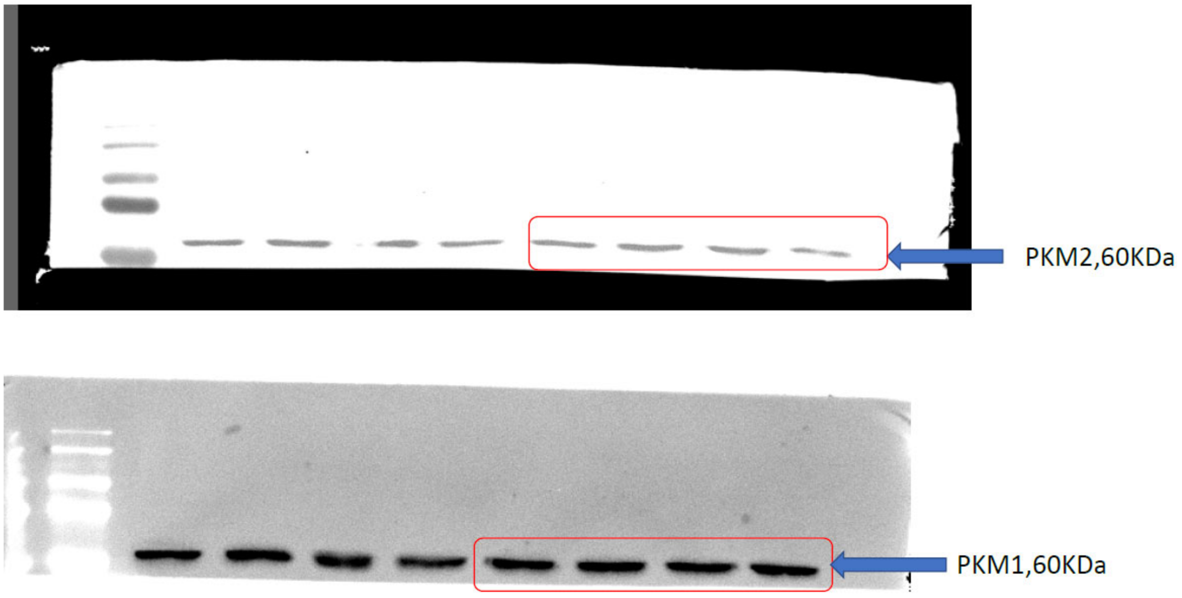

Figure S3. Raw data for Figure 4A.

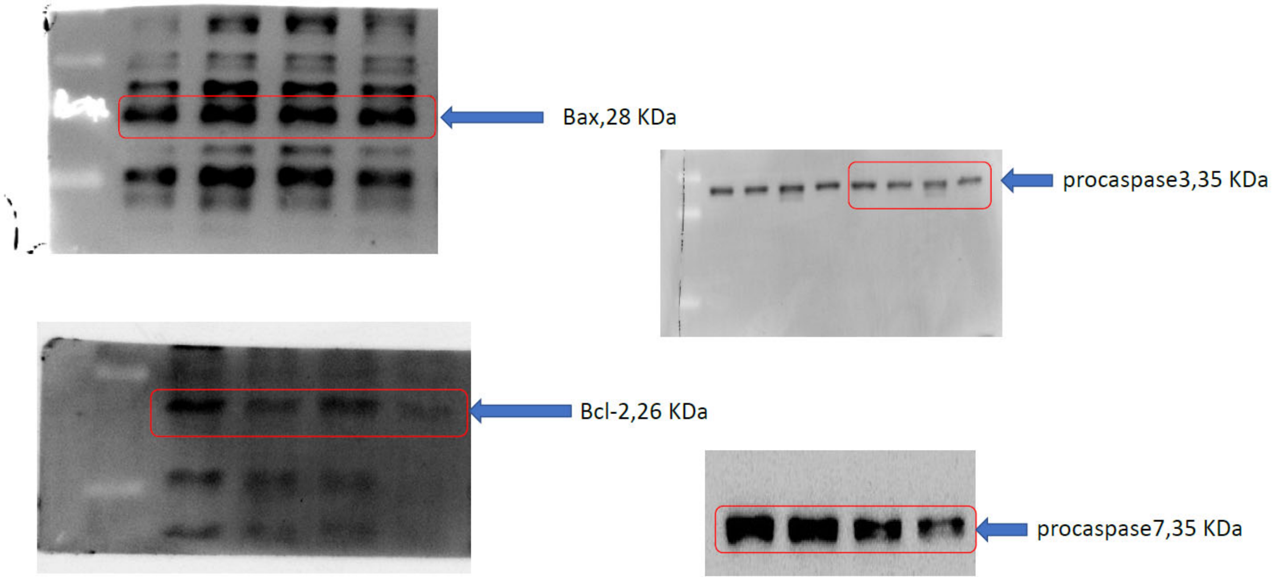

Figure S4. Raw data for Figure 6B.

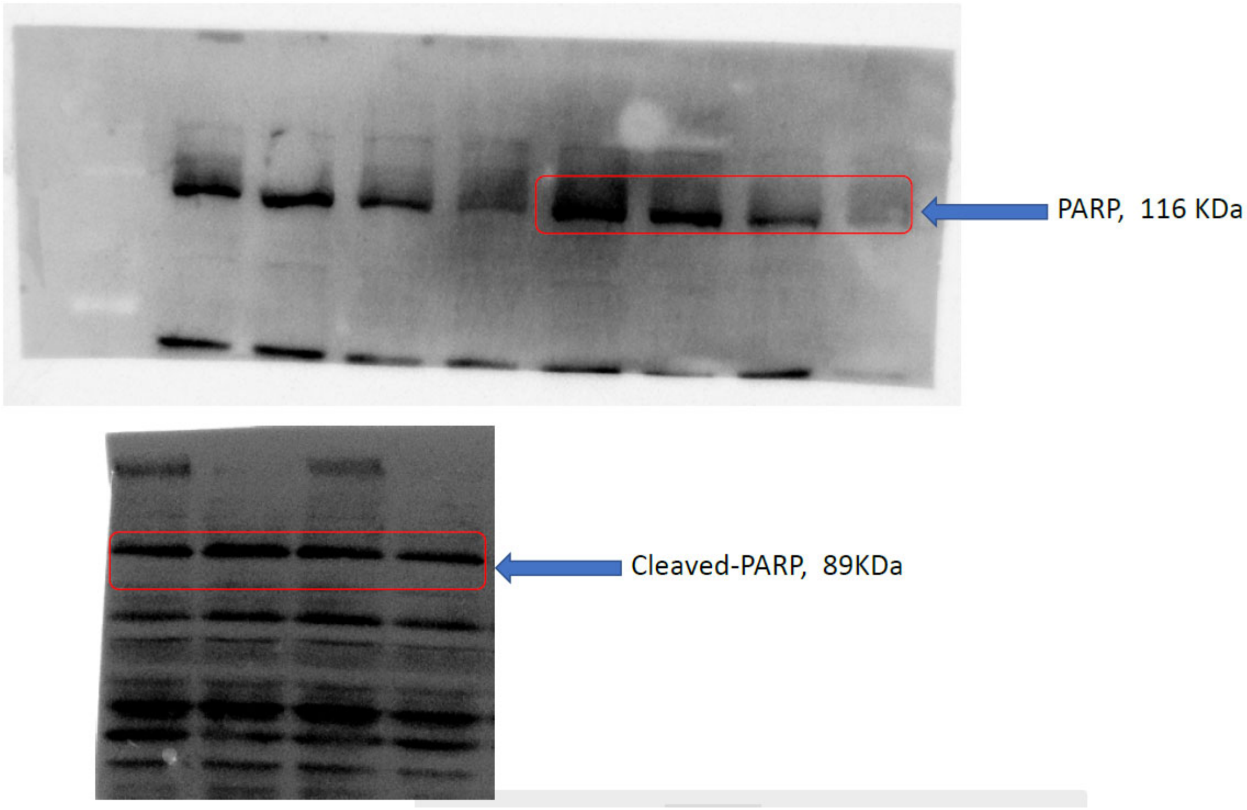

Figure S5. Raw data for Figure 6B.

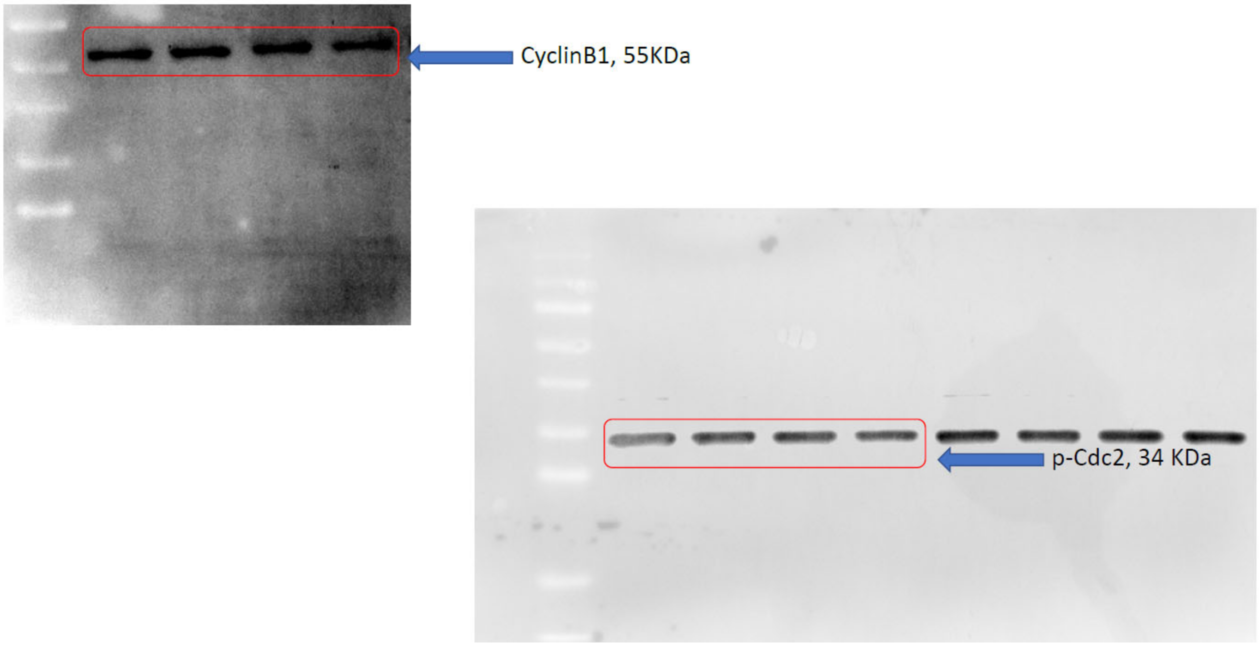

Figure S6. Raw data for Figure 7C.

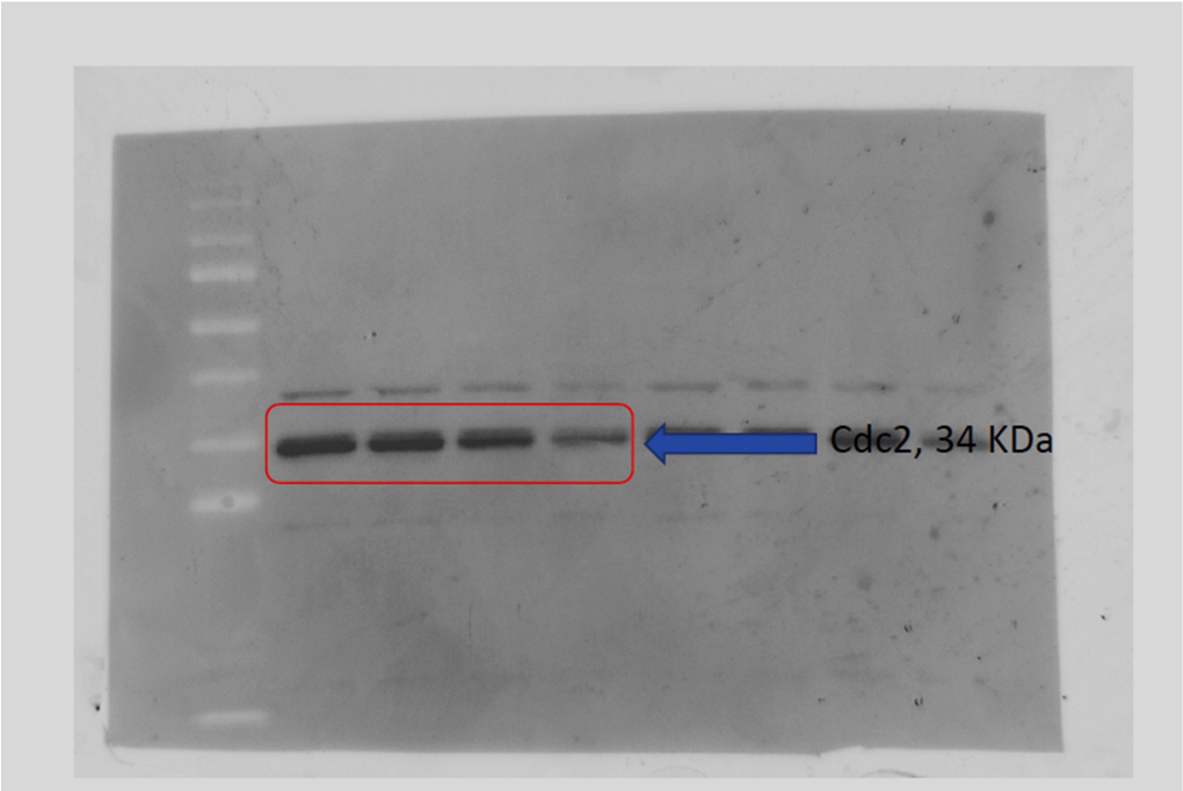

Figure S7. Raw data for Figure 7C.

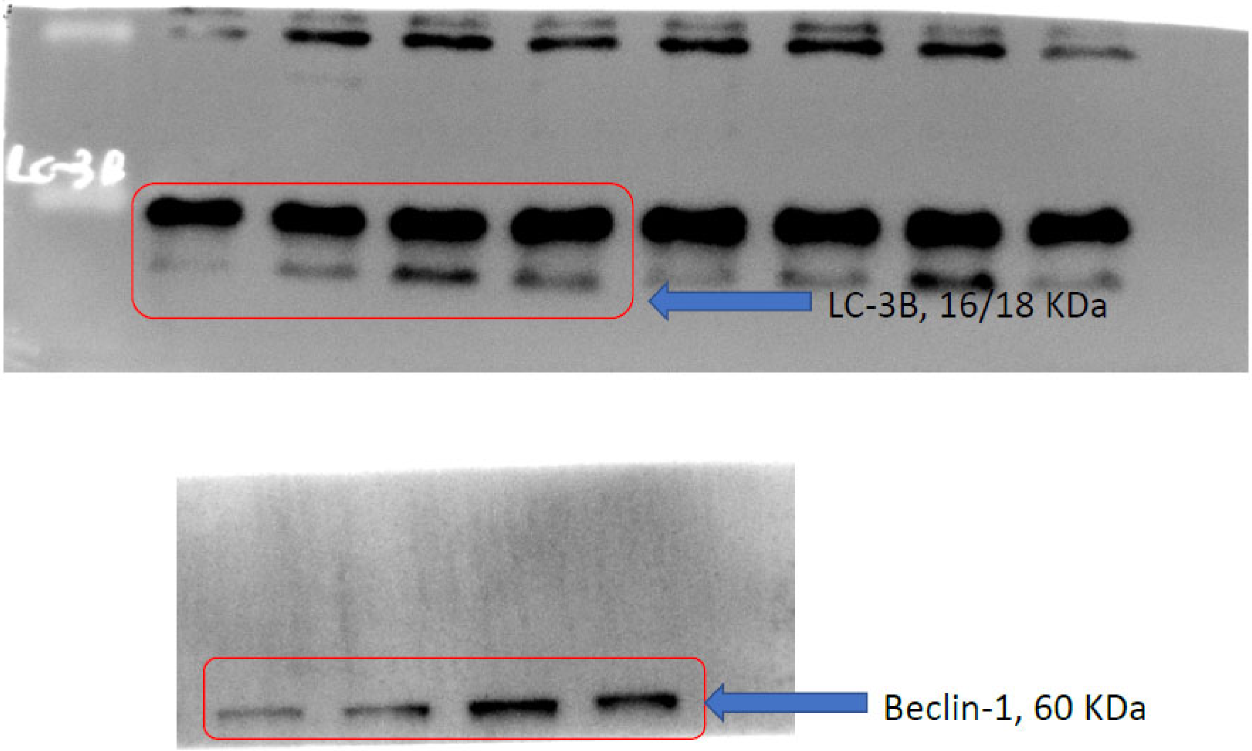

Figure S8. Raw data for Figure 7F.

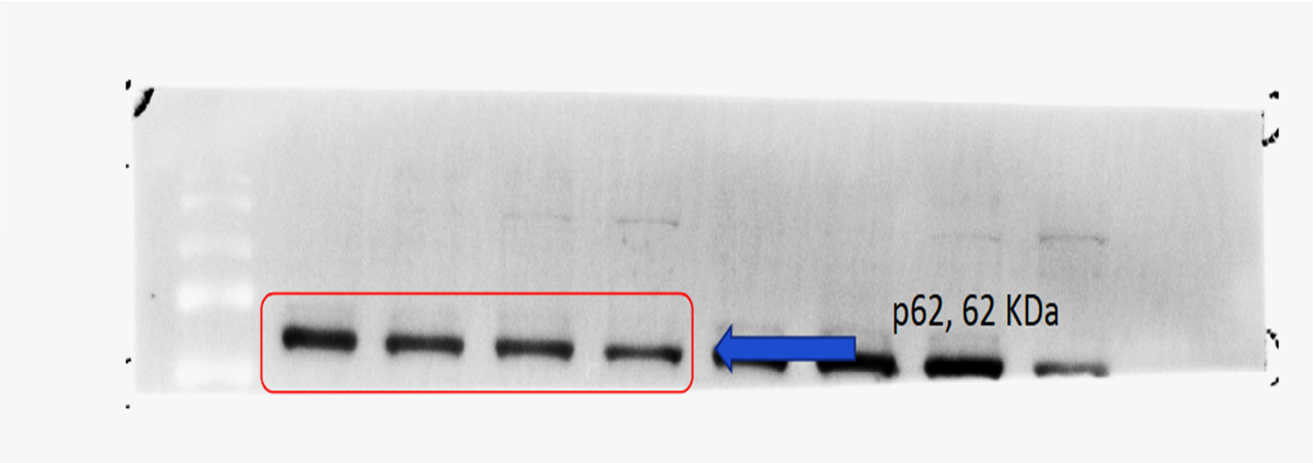

Figure S9. Raw data for Figure 7C.

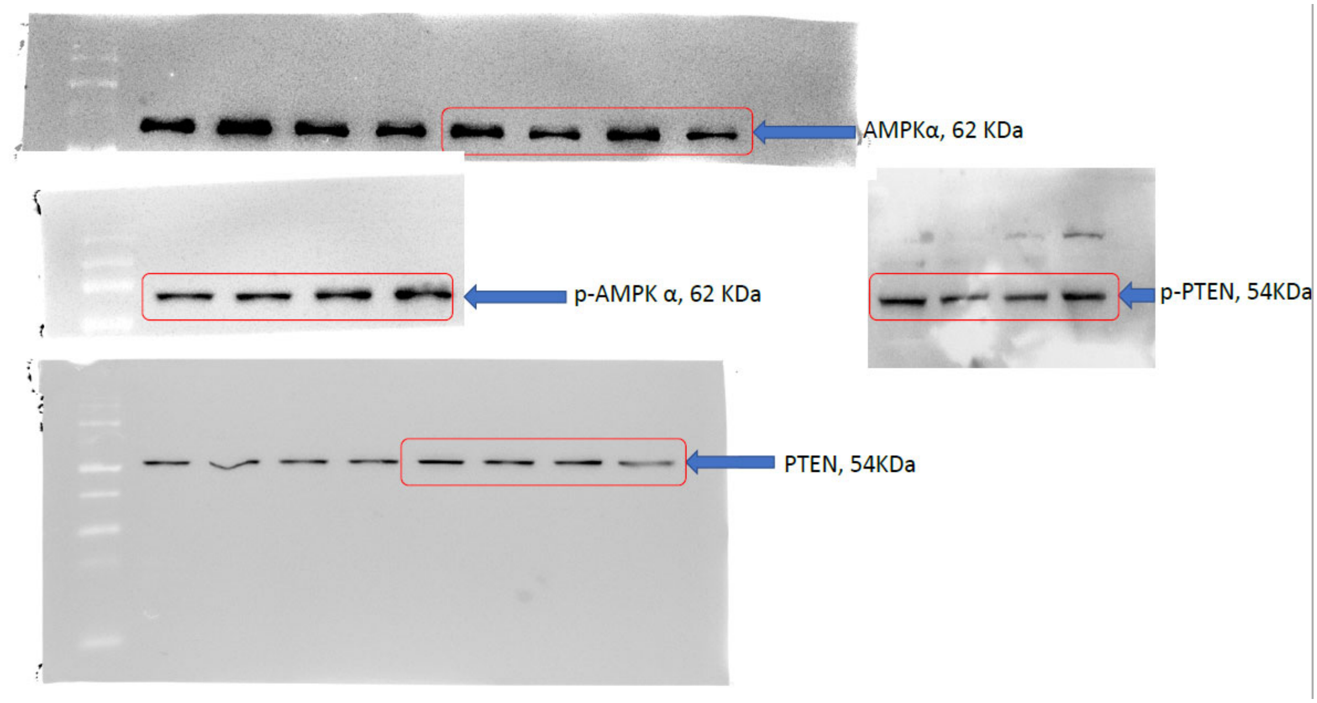

Figure S10. Raw data for Figure 8A.

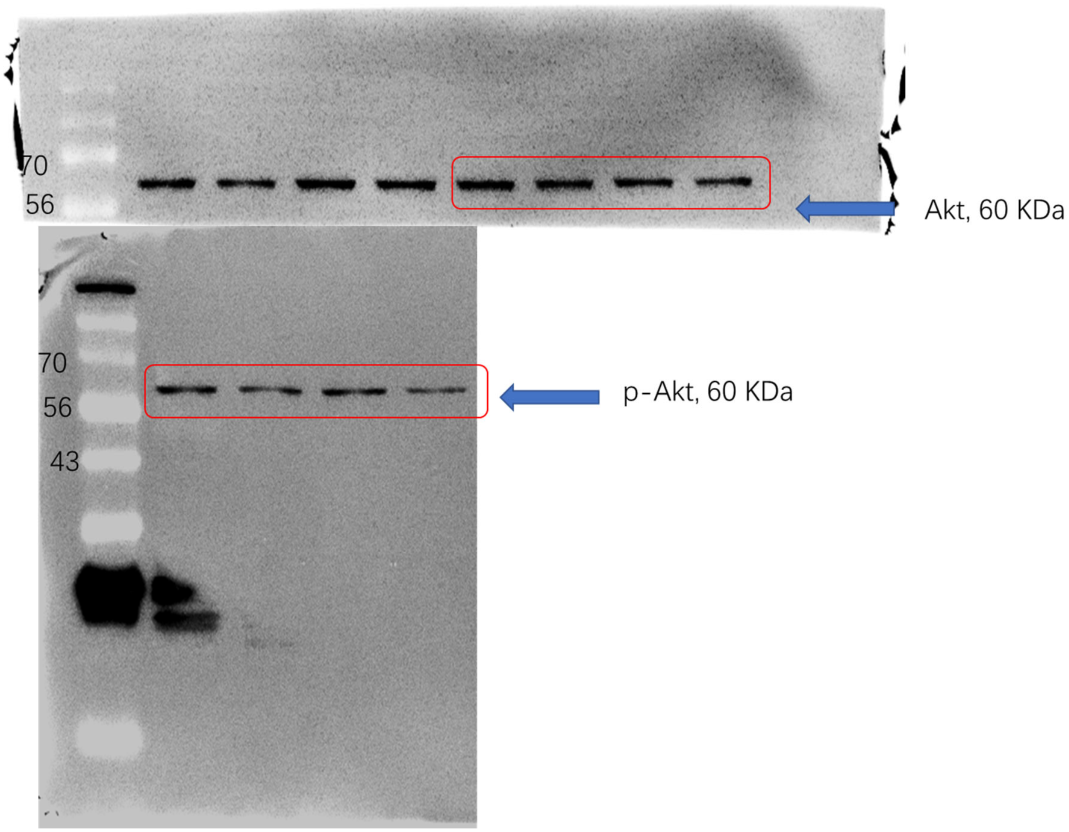

Figure S11. Raw data for Figure 8A.

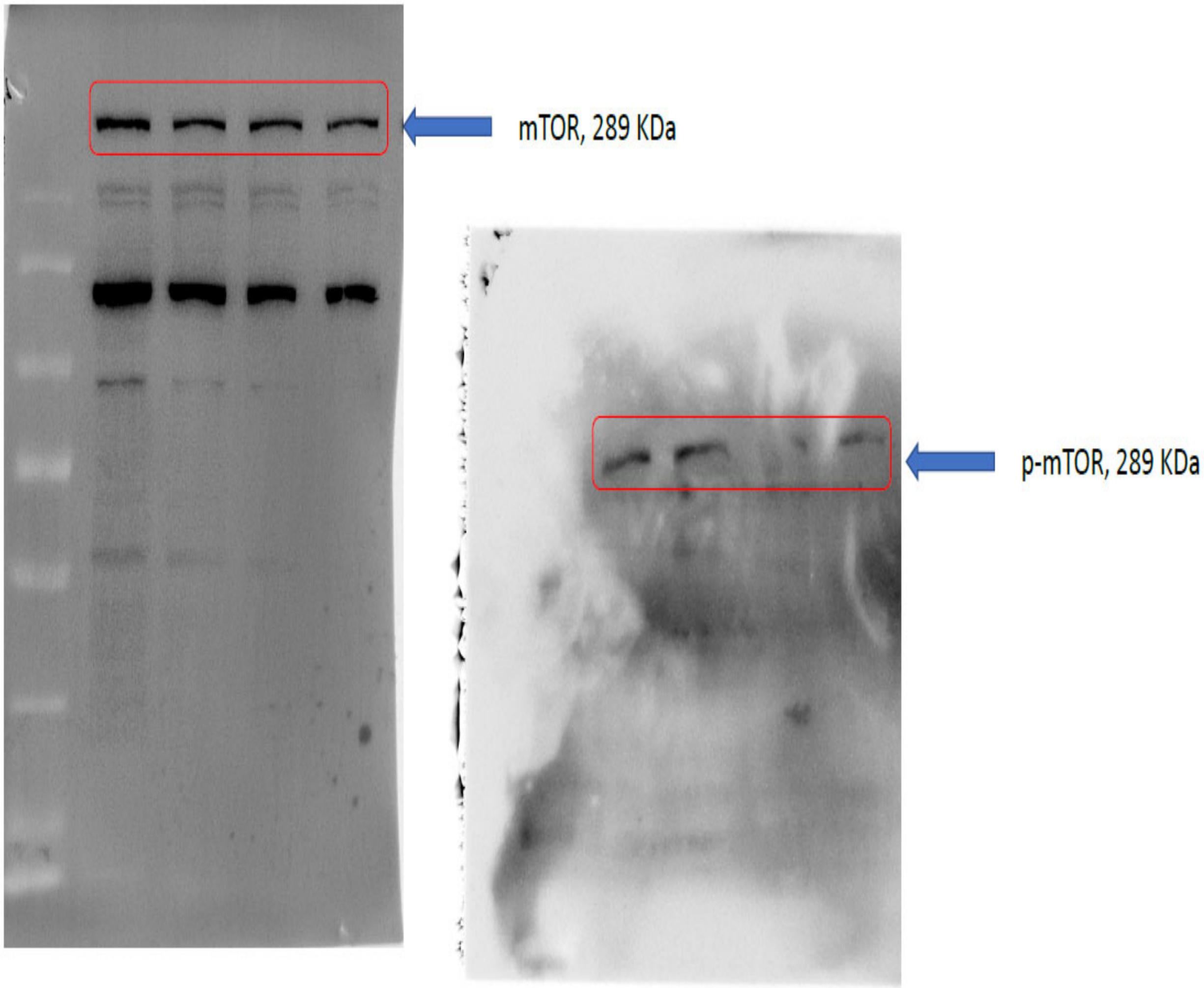

Figure S12. Raw data for Figure 8A.

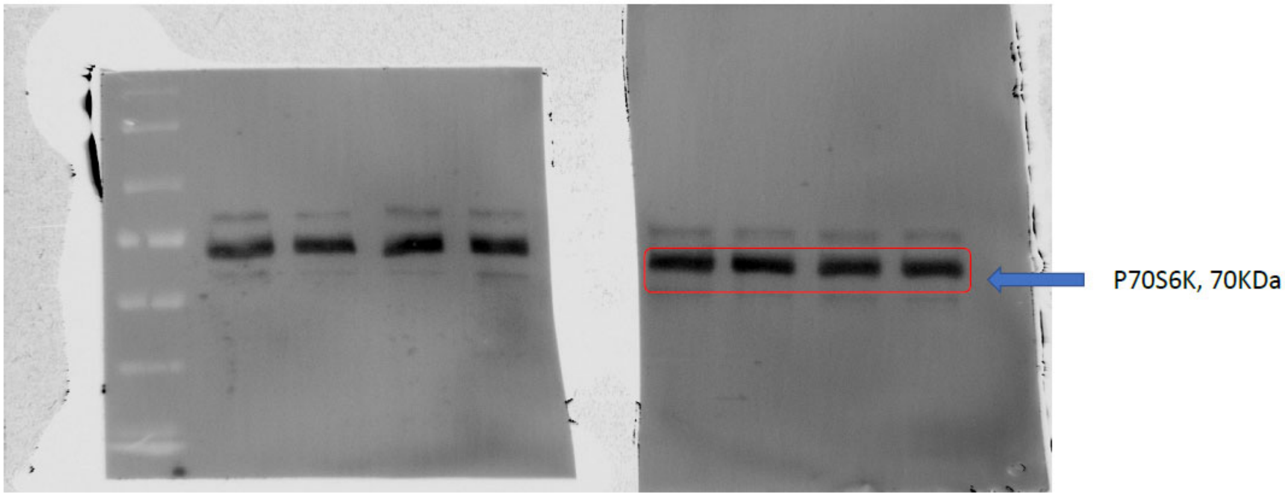

Figure S13. Raw data for Figure 8A.

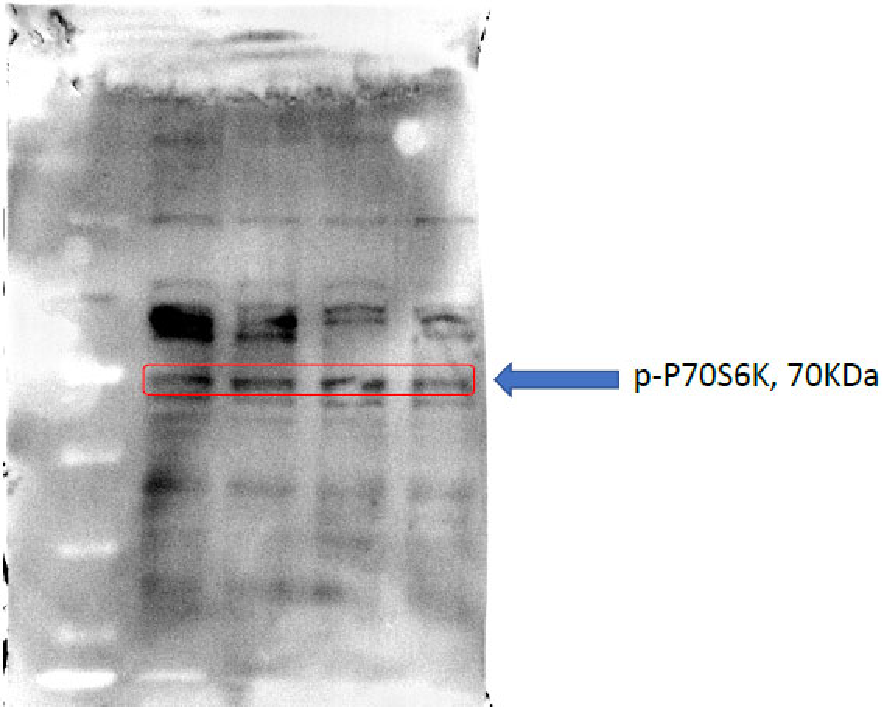

Figure S14. Raw data for Figure 8A.

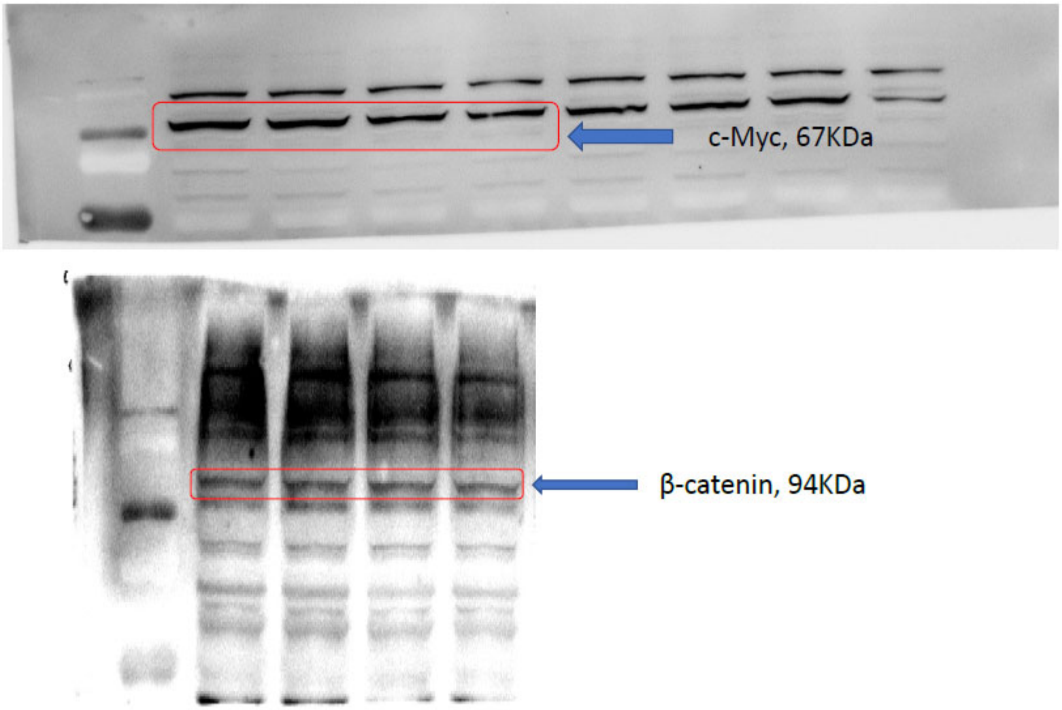

Figure S15. Raw data for Figure 10.

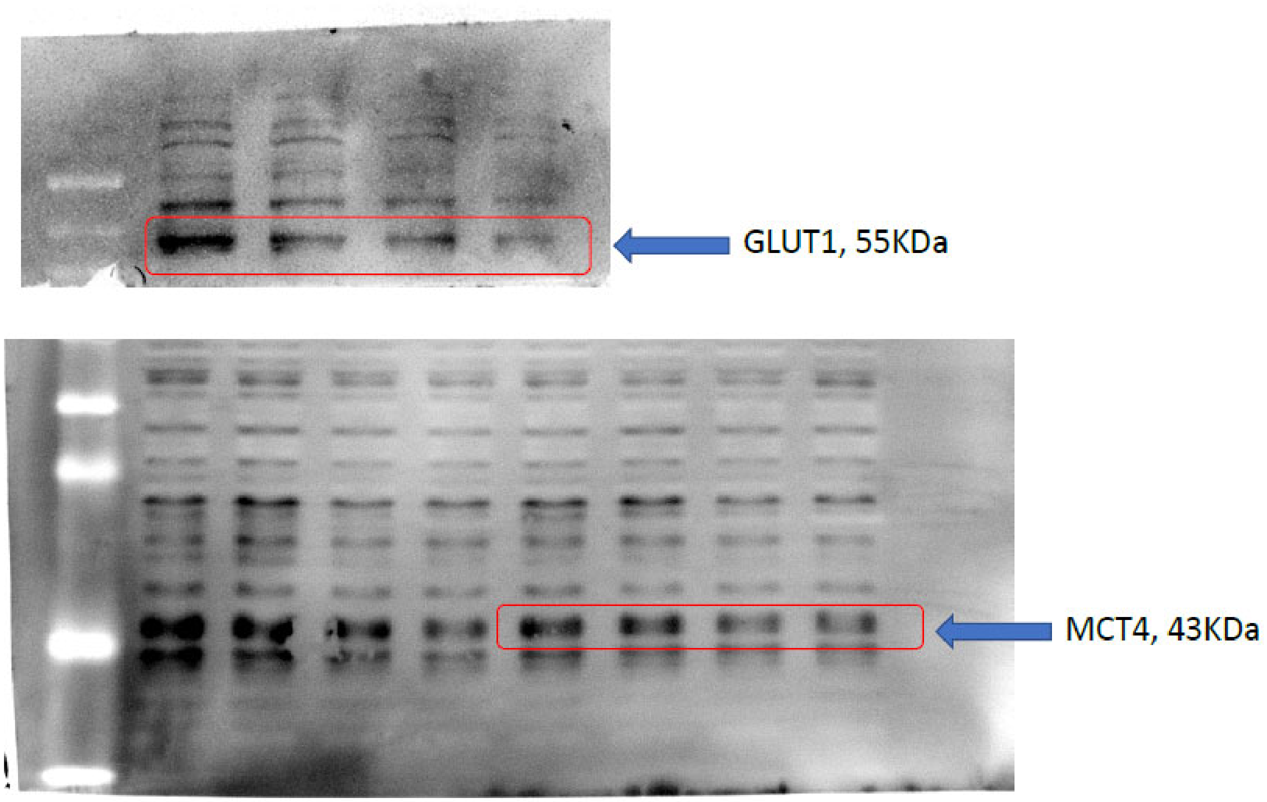

Figure S16. Raw data for Figure 10.
